# Fabrication of Z-scheme Ag_3_PO_4_/TiO_2_ Heterostructures for Enhancing Visible Photocatalytic Activity

**DOI:** 10.1186/s11671-019-3041-8

**Published:** 2019-06-13

**Authors:** Wenhui Liu, Dengdeng Liu, Kun Wang, Xiaodan Yang, Shuangqi Hu, Lishuang Hu

**Affiliations:** Environmental and Safety Engineering Institute, North University of China, Taiyuan, 030051 Shanxi People’s Republic of China

**Keywords:** Composite, Heterostructures, Superoxide anion, Photocatalytic degradation

## Abstract

In this paper, a synthetical study of the composite Ag_3_PO_4_/TiO_2_ photocatalyst, synthesized by simple two-step method, is carried out. Supplementary characterization tools such as X-ray diffraction, scanning electron microscopy, transmission electron microscopy, high-resolution transmission electron microscopy, energy dispersive X-ray spectroscopy, X-ray photoelectron spectroscopy, and UV-vis diffuse reflectance spectroscopy were adopted in this research. The outcomes showed that highly crystalline and good morphology can be observed. In the experiment of photocatalytic performance, TiO_2_400/Ag_3_PO_4_ shows the best photocatalytic activity, and the photocatalytic degradation rate reached almost 100% after illuminating for 25 min. The reaction rate constant of TiO_2_400/Ag_3_PO_4_ is the largest, which is 0.02286 min^−1^, twice that of Ag_3_PO_4_ and 6.6 times that of the minimum value of TiO_2_400. The degradation effect of TiO_2_400/Ag_3_PO_4_ shows good stability after recycling the photocatalyst four times. Trapping experiments for the active catalytic species reveals that the main factors are holes (h^+^) and superoxide anions (O·− 2), while hydroxyl radical (·OH) plays partially degradation. On this basis, a *Z*-scheme reaction mechanism of Ag_3_PO_4_/TiO_2_ heterogeneous structure is put forward, and its degradation mechanism is expounded.

## Background

Semiconductor photocatalysts have attracted increasing interest due to extenstive use in organic pollutant degradation and solar cells [1–6]. As the representative of semiconductor-based photocatalysts, TiO_2_ has been extensively investigated because of its excellent physical-chemical properties [7, 8]. However, the pure TiO_2_ photocatalyst has certain disadvantages in practical applications such as its wide band gap (3.2 eV for anatase and 3.0 eV for rutile), which leads to poor visible response.

A silver-based compound such as Ag_2_O, AgX (X = Cl, Br, I), Ag_3_PO_4_, Ag_2_CrO_4_, have been recently used for photocatalytic applications [9–12]. Among others, silver orthophosphate (Ag_3_PO_4_) has already attracted attention from many researchers because Ag_3_PO_4_ has a band gap of 2.45 eV and strong absorption at less than 520 nm. The quantum yield of Ag_3_PO_4_ is over 90%. It is a good visible-light photocatalyst. However, due to the formation of Ag^0^ on the surface of the catalyst (4Ag_3_PO_4_ + 6H_2_O + 12h^+^ + 12e^−^ → 12Ag^0^ + 4H_3_PO_4_ + 3O_2_) during the photocatalytic reaction, the reuse of Ag_3_PO_4_ is a major problem. Therefore, it is a common practice to reduce photocatalytic corrosion of Ag_3_PO_4_ and ensure good catalytic activity of Ag_3_PO_4_. Based on literature precedence, it is known that compounding can effectively improve the photocatalytic performance of both semiconductor materials. After compounding, the separation effect of photogenerated electrons and holes is strengthened, contributing to enhance the photocatalytic activity of composite materials. Numerous researchers have investigated heterojunctions such as Bi_2_O_3_-Bi_2_WO_6_, TiO_2_/Bi_2_WO_6_, ZnO/CdSe, and Ag_3_PO_4_/TiO_2_ [2, 13–15]. Compared with single-phase photocatalysts, heterojunction photocatalysts can expand the light response range by coupling matched electronic structure materials. And because of the synergistic effect between components, charge can be transferred through many ways to further improve heterojunction photocatalytic activity.

Based on the above analysis, Ag_3_PO_4_-based semiconductor composites with synergistic enhancement effect were designed to improve carrier recombination defects and Ag_3_PO_4_-based semiconductor composites catalytic performance. In this paper, nano-sized TiO_2_ was prepared by solvothermal method, and then the nanoparticles of TiO_2_400 were deposited on the surface of Ag_3_PO_4_ at room temperature to obtain TiO_2_/Ag_3_PO_4_ composites. The photocatalytic activity of TiO_2_/Ag_3_PO_4_ composite was tested using RhB dye (rhodamine B).

## Methods

### Hydrothermal Preparation of Nano-sized TiO_2_

0.4 g P123 was added to a mixed solution containing 7.6 mL absolute ethanol and 0.5 mL deionized water and stirred until P123 was completely dissolved. The clarified solution was labeled as A solution. Then a mixed solution containing 2.5 mL butyl titanate (TBOT) and 1.4 mL concentrated hydrochloric acid (12 mol/L) was prepared and labeled as B solution. The solution B was added to solution A by drop. After stirring for 30 min, 32 mL ethylene glycol (EG) was added to the solution and stirred for 30 min. Then, the solution was placed in oven, at 140 °C, high temperature, and high pressure for 24 h. Natural cooling, centrifugal washing, separation, collection of sediments, and drying at 80 °C oven for 8 h. The white precipitation was calcined in muffle furnace at different temperatures (300 °C, 400 °C, 500 °C) and marked as standby of TiO_2_300, TiO_2_400, and TiO_2_500, respectively.

### Preparation of TiO_2_/Ag_3_PO_4_ Photocatalyst

The 0.1 g TiO_2_ powder was added to the 30-mL silver nitrate solution containing 0.612 g AgNO_3_ and then treated by ultrasound for 30 min to make TiO_2_ dispersed uniformly. We added 30-mL solution containing 0.43 g Na_2_HPO_4_.12H_2_O and stirred for 120 min at ambient temperature. By centrifugation, cleaning with deionized water and anhydrous ethanol, the precipitates were separated, collected, and dried at 60 °C. The products were named as TiO_2_300/Ag_3_PO_4_, TiO_2_400/Ag_3_PO_4,_ and TiO_2_500/Ag_3_PO_4_, respectively. Ag_3_PO_4_ was prepared without adding TiO_2_ under the same conditions as the above process.

### Characterization

The X-ray diffraction (XRD) patterns of the resulted samples were performed on a D/MaxRB X-ray diffractometer (Japan), which has a 35 kV Cu-Ka with a scanning rate of 0.02° s^−1^, ranging from 10 to 80°. Scanning electron microscopy (SEM), JEOL, JSM-6510, and JSM-2100 transmission electron microscopy (TEM) assembly with energy dispersive X-ray spectroscopy (EDX) were used to study its morphology at 10-kV acceleration voltage. X-ray photoelectron spectroscopy (XPS) information were collected by using an ESCALAB 250 electron spectrometer under 300-W Cu Kα radiation. The basic pressure was about 3 × 10^−9^ mbar, Combine to refer to the C1s line at amorphous carbon 284.6 eV.

### Photocatalytic Activity Measure

The photocatalytic performance of TiO_2_/Ag_3_PO_4_ catalysts was tested by using the photodegradation of RhB in aqueous solution as the research object. Fifty milligrams of the photocatalyst was mixed with 50 mL of RhB aqueous solution (10 mg L^−1^) and stirred in darkness for a certain time before illumination to ensure adsorption balance. In the reaction process, cooling water is used to keep the system temperature constant at room temperature. A 1000-W Xenon lamp provides illumination to simulate visible light. LAMBDA35 UV/Vis spectrophotometer was used to characterize the concentration (*C*) change of RhB solution at *λ* = 553 nm. The decolorization rate is indicated as a function of time vs *C*_*t*_/*C*_0_. Where *C*_0_ is the concentration before illumination, and *C*_*t*_ is the concentration after illumination. Used catalysts were recollected to detect the cycle stability of the catalysts. The experiment was repeated four times.

## Results and Discussion

**Fig. 1:**
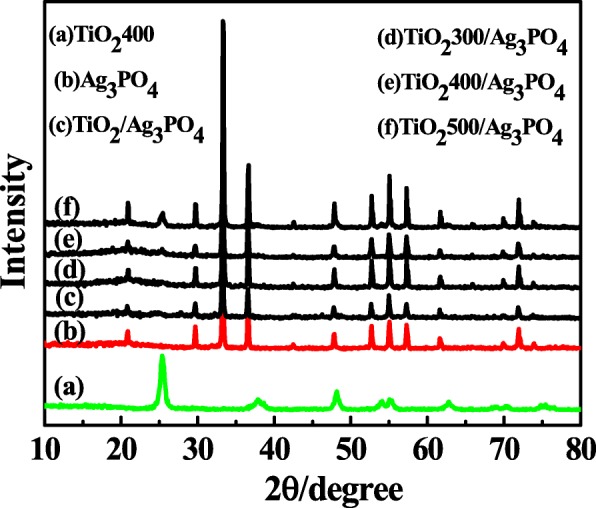
The XRD patterns of the as-prepared samples

XRD analysis is used to determine the phase structure and crystalline type of catalyst. The XRD spectra of the prepared catalysts were shown in Fig. 1, including TiO_2_400, Ag_3_PO_4_, TiO_2_/Ag_3_PO_4_, TiO_2_300/Ag_3_PO_4_, TiO_2_400/Ag_3_PO_4_, and TiO_2_500/Ag_3_PO_4_. It can be obtained from the figure that the crystal structure of TiO_2_400 is anatase (JCPDS No. 71-1166). In the XRD spectra of Ag_3_PO_4_, the diffraction peaks located at 20.9°, 29.7°, 33.3°, 36.6°, 47.9°, 52.7°, 55.1°, 57.4°, 61.7°, and 72.0° belong to the characteristic peaks of (110), (200), (210), (211), (310), (222), (320), (321), (400), and (421) planes of Ag_3_PO_4_ (JCPDS No. 70-0702), respectively. The synthesized composite photocatalysts showed characteristic peaks consistent with TiO_2_ and Ag_3_PO_4_, and the characteristic peaks of TiO_2_ were 25.3° at the composite TiO_2_, TiO_2_300/Ag_3_PO_4_, TiO_2_400/Ag_3_PO_4_, TiO_2_500/Ag_3_PO_4_, which was consistent with the calcination temperature of TiO_2_ rise, the crystallinity of TiO_2_ becomes higher.

**Fig. 2:**
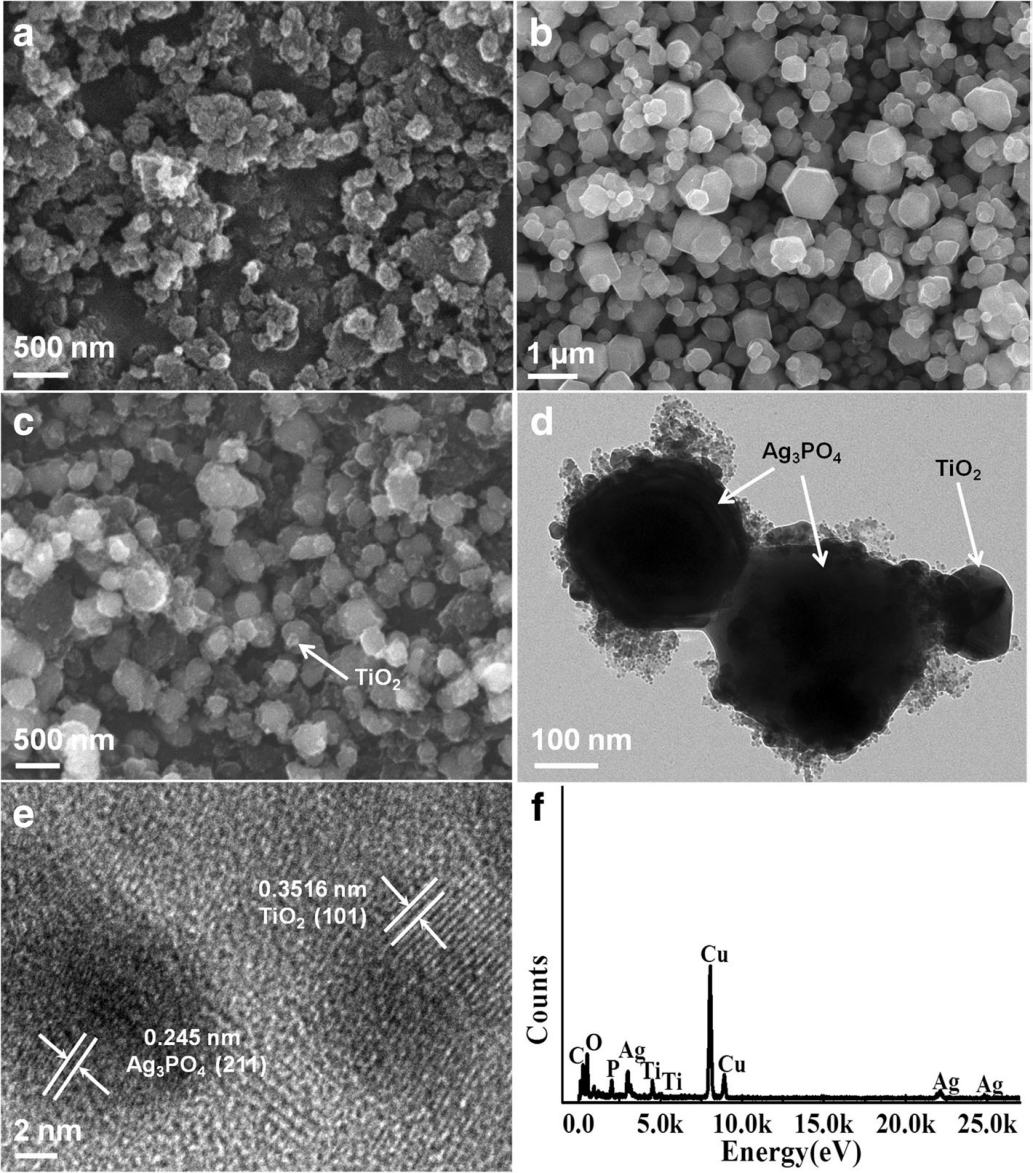
SEM images of prepared photocatalysts: **a** TiO_2_400, **b** Ag_3_PO_4_, **c** TiO_2_400/Ag_3_PO_4_, **d** TEM image of TiO_2_400/Ag_3_PO_4_, **e** HRTEM image of TiO_2_400/Ag_3_PO_4_, and **f** corresponding EDX pattern of TiO_2_400/Ag_3_PO_4_

Figure 2 shows the SEM, TEM, and EDX diagrams of the catalysts of TiO_2_400, Ag_3_PO_4_, and TiO_2_400/Ag_3_PO_4_. Figure 2a is the spherical nanostructure TiO_2_400 prepared by solvothermal method with a diameter ranging from 100 to 300 nm. Figure 2b is the Ag_3_PO_4_ crystal with a regular hexahedral structure. Its particle size ranges from 0.1 to 1.5 μm and has a fairly smooth surface. Figure 2c is the SEM image of the composite TiO_2_400/Ag_3_PO_4_. It can be seen that the nanoparticles of TiO_2_400 are deposited on the surface of Ag_3_PO_4_. The morphology of TiO_2_400/Ag_3_PO_4_ was further explored with TEM and the TEM diagram of TiO_2_400/Ag_3_PO_4_ is displayed in Fig. 2d. It can be observed that 200-nm nano-sized TiO_2_ particles adhere to the surface of Ag_3_PO_4_. Figure 2e is the HRTEM of TiO_2_400/Ag_3_PO_4_. It can be founded that TiO_2_ particles are closely bound to Ag_3_PO_4_, and the lattice spacing of TiO_2_400 and Ag_3_PO_4_ are 0.3516 and 0.245 nm, respectively, corresponding to (101) and (211) surfaces of TiO_2_ and Ag_3_PO_4_. Figure 2f is the EDX diagram of TiO_2_400/Ag_3_PO_4_. It can be seen that the sample consists of four elements: Ti, O, Ag, and P. The obvious diffraction peak of copper element is produced by the EDX excitation source, Cu Ka. EDX confirmed the corresponding chemical elements of TiO_2_400/Ag_3_PO_4_. In conclusion, it can be clearly judged that TiO_2_ is loaded on the surface of Ag_3_PO_4_ crystals in granular form and has a good hexahedron morphology.

**Fig. 3:**
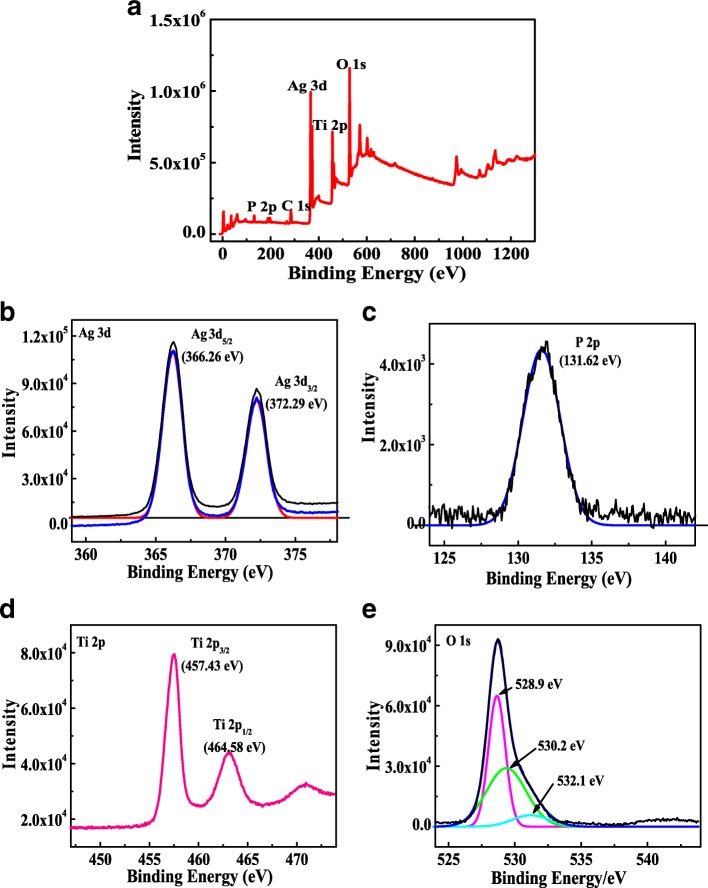
XPS spectrum of TiO_2_400/Ag_3_PO_4_: **a** survery scan, **b** Ag 3d, **c** P 2p, **d** Ti 2p, and **e** O1s

The product X-ray photoelectron spectroscopy (XPS) is investigated in Fig. 3. Figure 3a is the survey XPS spectrum of the product. Ti, O, Ag, P, and C five elements can be observed in the graph, of which C is the base, implying that composite coexisted with TiO_2_ and Ag_3_PO_4_. Figure 3b is the high-resolution spectrum of Ag 3d. The two main peaks centered at binding energy 366.26 eV and 372.29 eV, assigning to Ag 3d5/2 and Ag 3d3/2, respectively. It shows that Ag is mainly Ag^+^ in the photocatalyst of TiO_2_400/Ag_3_PO_4_ [16]. Figure 3c shows the XPS peak of P 2p, which corresponds to P^5+^ in the PO_4_^3+^ structure at 131.62 eV. Two peaks located at 457.43 eV and 464.58 eV can be attributed to Ti 2p3/2 and Ti 2p1/2 in the XPS spectrum of Ti 2p orbital (Fig. 3d). Figure 3e is the XPS of O 1s. The whole peak can be divided into three characteristic peaks, 528.9 eV, 530.2 eV, and 532.1 eV. The peaks at 528.9 eV and 530.2 eV are ascribed to oxygen in Ag_3_PO_4_ and TiO_2_ lattices, respectively. The peaks at 532.1 eV indicate hydroxyls or the oxygen adsorbed on the surface of TiO_2_/Ag_3_PO_4_. The results of XPS analysis further prove that Ag_3_PO_4_ and TiO_2_ have been compounded.

**Fig. 4:**
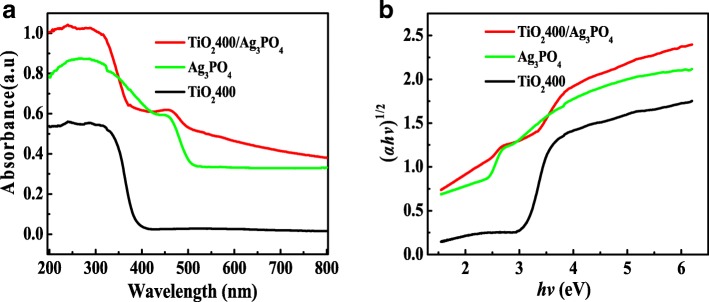
TiO_2_400, Ag_3_PO_4_, and TiO_2_400/Ag_3_PO_4_ catalysts: **a** UV-Vis DRS, **b** plots of (*α*hv)^1/2^ versus energy (hv)

The UV-Vis diffuse reflectance absorption spectra of the catalysts of TiO_2_400, Ag_3_PO_4_, and TiO_2_400/Ag_3_PO_4_ are exhibited in Fig. 4a. It can be seen from the figure that the optical absorption cutoff wavelengths of TiO_2_400 and Ag_3_PO_4_ are 400 and 500 nm, respectively. When Ag_3_PO_4_ is loaded on TiO_2_400, the light absorption range of the composite obviously broadens to 500–700 nm, indicating that there is interaction between Ag_3_PO_4_ and TiO_2_400 in the composite system of TiO_2_400/Ag_3_PO_4_, and the mechanism needs further study. Bandwidth of Ag_3_PO_4_, TiO_2_400, and TiO_2_400/Ag_3_PO_4_ catalysts is computed with the Kubelka-Munk formula [17]:

$$ A\mathrm{hv}=c{\left(\mathrm{hv}-\mathrm{Eg}\right)}^n $$

where *A*, hv, *c*, and Eg are the absorption coefficient, incident photon energy, absorption constant, and band gap energy, respectively. The value of *n* for direct semiconductor is 1/2, and that for indirect semiconductor is 2. Anatase TiO_2_ and Ag_3_PO_4_ are indirect semiconductors, so *n* takes 2.

The plots depicting (*α*hv)^1/2^ versus incident photon energy (hv) from Fig. 4b indicates the band gap energy diagrams (Eg) of Ag_3_PO_4_, TiO_2_400, and TiO_2_400/Ag_3_PO_4_ catalysts are 2.45 eV, 3.1 eV, and 2.75 eV, respectively. This further proves that TiO_2_400/Ag_3_PO_4_ is a good visible-light photocatalyst with suitable band gap width and visible light capture ability.

**Fig. 5:**
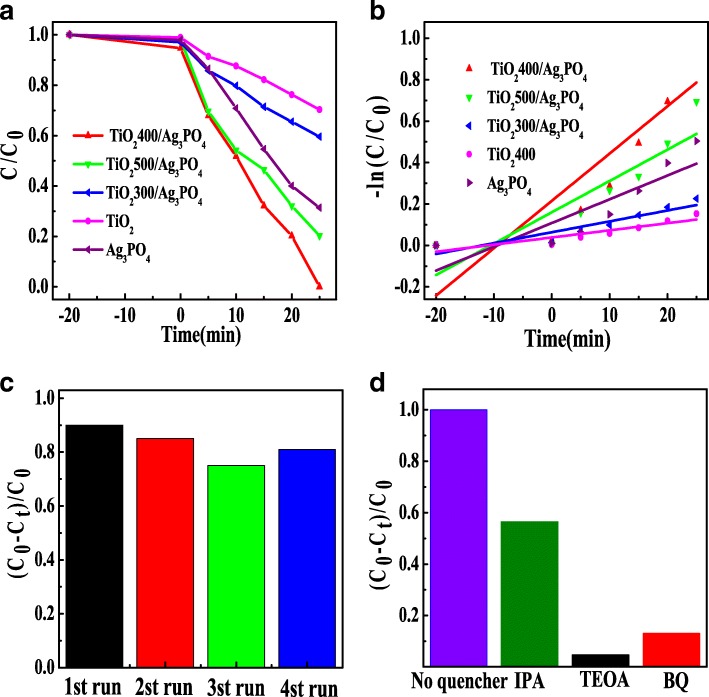
**a** Effects of different catalysts on photocatalytic degradation of RhB under visible light. **b** First order kinetic fitting plots of photocatalytic degradation of RhB with different catalysts. **c** Cycling runs of TiO_2_400/Ag_3_PO_4_. **d** Trapping experiments of active species

Photocatalytic degradation of RhB by TiO_2_400, Ag_3_PO_4_, TiO_2_300/Ag_3_PO_4_, TiO_2_400/Ag_3_PO_4_, and TiO_2_500/Ag_3_PO_4_ was investigated in Fig. 5a. The results showed that pure TiO_2_400 had the worst photocatalytic effect, and the photocatalytic degradation rate was only 30% within 25 min. The photocatalytic degradation efficiency of pure Ag_3_PO_4_ was 69% after 25 min of irradiation. The photocatalytic degradation rate of TiO_2_300/Ag_3_PO_4_ reached 40% after 25 min. The photocatalytic degradation rate of TiO_2_500/Ag_3_PO_4_ was 80% after 25 min of irradiation. The best photocatalytic activity was TiO_2_400/Ag_3_PO_4_, and 100% of RhB was decomposed after 25 min of illumination.

Figure 5b studied the kinetics model of photocatalytic degradation of RhB. From the figure, the photodegradation of RhB was followed pseudo-first-order kinetics and the reaction rate constant (*k*) was calculated with the slope of fitting curves. The reaction rate constant (*k*) values of each sample were shown in Table 1. The reaction rate constants of TiO_2_400, Ag_3_PO_4_, TiO_2_300/Ag_3_PO_4_, TiO_2_400/Ag_3_PO_4_, and TiO_2_500/Ag_3_PO_4_ were 0.00345 min^−1^, 0.01148 min^−1^, 0.00525 min^−1^, 0.02286 min^−1^, and 0.01513 min^−1^, respectively. The sample TiO_2_400/Ag_3_PO_4_ has the largest reaction rate constant, which is 0.02286 min^−1^, twice that of Ag_3_PO_4_ and 6.6 times that of the minimum value of TiO_2_400. This indicates that the combination of Ag_3_PO_4_ and TiO_2_ can greatly contribute to the improvement of Ag_3_PO_4_ photocatalytic activity.

**Table 1:** Photo degradation rate constants and linear regression coefficients of different catalysts from equation − ln(*C*/*C*_0_) = kt.

	*K* (min^−1^)	Regression equation	*R* ^2^
TiO_2_400/Ag_3_PO_4_	0.02286	− ln(*C*/*C*_0_) = 0.02286*x* + 0.21496	*R*^2^ = 0.68755
TiO_2_500/Ag_3_PO_4_	0.01513	− ln(*C*/*C*_0_) = 0.01513*x* + 0.15984	*R*^2^ = 0.753
Ag_3_PO_4_	0.01148	− ln(*C*/*C*_0_) = 0.01148*x* + 0.1079	*R*^2^ = 0.71128
TiO_2_300/Ag_3_PO_4_	0.00525	− ln(*C*/*C*_0_) = 0.00525*x* + 0.06354	*R*^2^ = 0.82635
TiO_2_400	0.00345	− ln(*C*/*C*_0_) = 0.00345*x* + 0.0383	*R*^2^ = 0.78461

Figure 5c is the stability test result of four times of degradation of RhB solution by recycling of TiO_2_400/Ag_3_PO_4_. The degradation effect of TiO_2_400/Ag_3_PO_4_ shows good stability in four times of recycling, and in the fourth cycle experiment, the degradation effect of TiO_2_400/Ag_3_PO_4_ was slightly higher than that of the third cycle. This may be due to the formation of composite material between Ag_3_PO_4_ and TiO_2_ to accelerate photogenerated electron-hole pair transfer and in situ formation of a small amount of Ag in Ag_3_PO_4_ during photocatalysis to inhibit further photo-corrosion.

The results of TiO_2_/Ag_3_PO_4_ capture factors are shown in Fig. 5d. After the addition of trapping agent IPA, the degradation activity decreased partially. When BQ and TEOA were added, the degradation degree of RhB decreased significantly, even close to 0. Therefore, we can infer that the main factors are holes (h^+^) and superoxide anions (O·− 2), while hydroxyl radical (·OH) plays partially degradation.

**Scheme 1:**
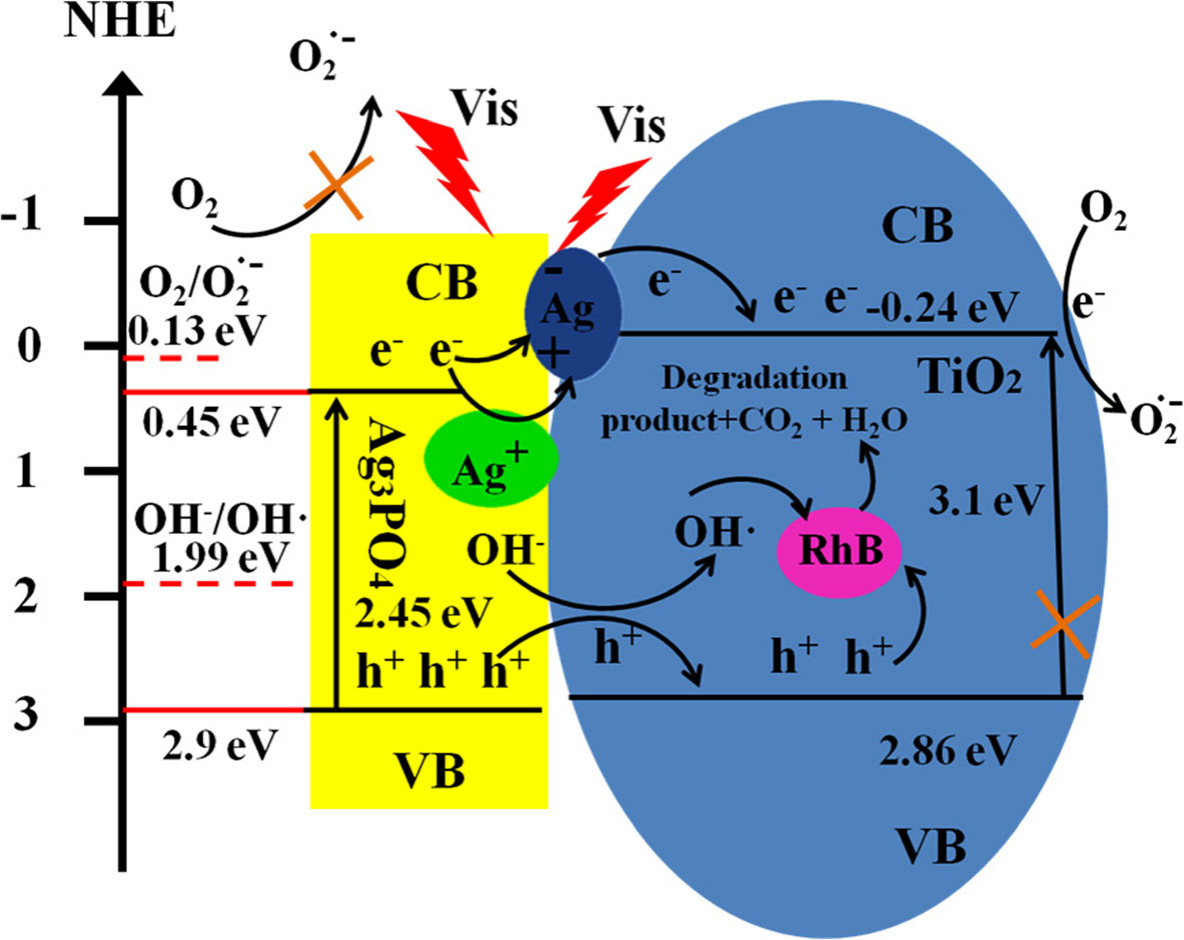
Schematic illustration of the photocatalytic mechanism of TiO_2_/Ag_3_PO_4_

A possible Z-scheme photocatalytic degradation mechanism was proposed in Scheme 1 to expatiate the photocatalytic degradation of RhB by TiO_2_/Ag_3_PO_4_ based on free radical capture and photodegradation experiments. The band gap of Ag_3_PO_4_ is 2.45 eV, and its *E*_CB_ and *E*_VB_ potential are ca.0.45 eV and 2.9 eV (vs. NHE) [18], respectively. As shown in Scheme 1, under visible light irradiation, Ag_3_PO_4_ is stimulated by photons with energy greater than its band gap to produce photogenerated electron-hole pairs. The holes left in the valence band of Ag_3_PO_4_ migrated to the valence band of TiO_2_ and then directly participated in the RhB oxidation and decomposition process, which adsorbed on the surface of TiO_2_. At the same time, during the migration of photogenerated holes, the H_2_O and OH^−^ adsorbed on the composite surface can also be oxidized to form ·OH, and the highly oxidizing ·OH can further oxidize and degrade pollutants. This is mainly due to the energy of holes in the valence band of Ag_3_PO_4_ which is 2.9 eV, higher than the reaction potential energy of OH^−^/OH (E(OH^−^/OH) = 1.99 eV (vs. NHE)). However, the conduction potential of Ag_3_PO_4_ is 0.45 eV, the energy of photogenerated electrons is 0.45 eV, and the activation energy of single electron oxygen is E(O_2_/O·− 2) = 0.13 eV (vs. NHE). The photogenerated electrons on Ag_3_PO_4_ conduction band cannot be captured by dissolved oxygen. With the accumulation of photogenerated electrons on Ag_3_PO_4_ conductive band, a small amount of Ag nanoparticles has been formed due to the photocatalytic corrosion of Ag_3_PO_4_ photocatalyst. The formed Ag nanoparticles can also be stimulated by light energy to form photogenerated electron-hole pairs. Then the electrons migrated to the conduction band of TiO_2_, while the holes left on the Ag nanoparticles can be compounded with the photogenerated electrons generated on the conduction band of Ag_3_PO_4_, thus preventing the further corrosion of Ag_3_PO_4_ photocatalyst. Due to the forbidden band of TiO_2_ is 3.1 eV, it cannot be excited under visible light and the *E*_CB_ and *E*_VB_ are ca. − 0.24 eV and 2.86 eV (vs. NHE), respectively. Electrons injected into TiO_2_ conduction band can degrade pollutants through trapping the oxygen adsorbed onto the TiO_2_ surface. This is mainly due to the *E*_CB_ = − 0.24 eV (vs. NHE) which is more negative than E(O_2_/O·- 2) = 0.13 eV (vs. NHE). The results are in accordance with the trapping experiments. The main factors are holes (h^+^) and superoxide anions (O·- 2), while hydroxyl radical (·OH) plays partially degradation.

Basing on the above discussion, the degradation reaction of TiO_2_/Ag_3_PO_4_ is expressed by the chemical equation as follows:

Generation of photoelectron hole pairs:

$$ {\mathrm{Ag}}_3\mathrm{P}{\mathrm{O}}_4+\mathrm{hv}\to {\mathrm{Ag}}_3\mathrm{P}{\mathrm{O}}_4\left({\mathrm{e}}^{-}\right)+{\mathrm{Ag}}_3\mathrm{P}{\mathrm{O}}_4\left({\mathrm{h}}^{+}\right) $$

$$ {\mathrm{Ag}}^{+}+{\mathrm{Ag}}_3\mathrm{P}{\mathrm{O}}_4\left({\mathrm{e}}^{-}\right)\to \mathrm{Ag}+{\mathrm{Ag}}_3\mathrm{P}{\mathrm{O}}_4 $$

$$ \mathrm{Ag}+\mathrm{hv}\to \mathrm{Ag}\left({\mathrm{e}}^{-}\right)+\mathrm{Ag}\left({\mathrm{h}}^{+}\right) $$

Migration and transformation of photogenerated hole electron pairs:

$$ {\mathrm{Ag}}_3\mathrm{P}{\mathrm{O}}_4\left({\mathrm{h}}^{+}\right)+\mathrm{Ti}{\mathrm{O}}_2\to \mathrm{Ti}{\mathrm{O}}_2\left({\mathrm{h}}^{+}\right)+{\mathrm{Ag}}_3\mathrm{P}{\mathrm{O}}_4 $$

$$ {\mathrm{Ag}}_3\mathrm{P}{\mathrm{O}}_4\left({\mathrm{e}}^{-}\right)+\mathrm{Ag}\left({\mathrm{h}}^{+}\right)\to \mathrm{Ag}+{\mathrm{Ag}}_3\mathrm{P}{\mathrm{O}}_4 $$

$$ \mathrm{Ag}\left({\mathrm{e}}^{-}\right)+\mathrm{Ti}{\mathrm{O}}_2\to \mathrm{Ti}{\mathrm{O}}_2\left({\mathrm{e}}^{-}\right)+\mathrm{Ag} $$

$$ \mathrm{Ti}{\mathrm{O}}_2\left({\mathrm{e}}^{-}\right)+{\mathrm{O}}_2\to {\mathrm{O}}_2^{\cdotp -}+\mathrm{Ti}{\mathrm{O}}_2 $$

$$ {\mathrm{Ag}}_3\mathrm{P}{\mathrm{O}}_4\left({\mathrm{h}}^{+}\right)+0{\mathrm{H}}^{-}\to \mathrm{OH}\cdotp +{\mathrm{Ag}}_3\mathrm{P}{\mathrm{O}}_4 $$

Degradation of pollutants:

$$ \mathrm{Ti}{\mathrm{O}}_2\left({\mathrm{h}}^{+}\right)+\mathrm{RhB}\to \mathrm{Degradation}\ \mathrm{product}+{\mathrm{CO}}_2+{\mathrm{H}}_2\ \mathrm{O} $$

$$ {\mathrm{O}}_2^{\cdotp -}+\mathrm{RhB}\to \mathrm{Degradation}\ \mathrm{product}+{\mathrm{CO}}_2+{\mathrm{H}}_2\ \mathrm{O} $$

$$ \mathrm{OH}\cdotp +\mathrm{RhB}\to \mathrm{Degradation}\ \mathrm{product}+{\mathrm{CO}}_2+{\mathrm{H}}_2\ \mathrm{O}+{\mathrm{Cl}}^{-} $$

## Conclusions

In summary, a comprehensive investigation of the composite Ag_3_PO_4_/TiO_2_ photocatalyst, prepared by a simple two-step method is presented. Complementary characterization tools such as X-ray diffraction (XRD), scanning electron microscopy (SEM), transmission electron microscopy (TEM), high-resolution transmission electron microscopy (HR-TEM), energy dispersive X-ray spectroscopy (EDX), X-ray photoelectron spectroscopy (XPS), and UV-vis diffuse reflectance spectroscopy (DRS) were utilized in this study. The results showed that the composite Ag_3_PO_4_/TiO_2_ photocatalyst is highly crystalline and has good morphology. For Ag_3_PO_4_/TiO_2_ degradation of RhB, TiO_2_400/Ag_3_PO_4_ shows the highest photocatalytic activity. After 25 min of reaction, the photocatalytic degradation rate reached almost 100%. The reaction rate constant of TiO_2_400/Ag_3_PO_4_ is 0.02286 min^−1^, which is twice that of Ag_3_PO_4_ and 6.6 times that of the minimum value of TiO_2_400. The TiO_2_400/Ag_3_PO_4_ also exhibits good stability after recycling four times. The main active catalytic species are holes (h^+^) and superoxide anions (O·− 2), while hydroxyl radical (·OH) plays partially degradation from trapping experiments. In addition, a Z-scheme reaction mechanism of Ag_3_PO_4_/TiO_2_ heterogeneous structure is proposed to explain the RhB degradation mechanism. The accumulation of photogenerated electrons on Ag_3_PO_4_ conductive band causes photoetching of Ag_3_PO_4_ photocatalyst to form a small amount of Ag nanoparticles, consequently, accelerating photogenerated electron transfer in the Ag_3_PO_4_ conduction band, thus preventing further Ag_3_PO_4_ photocatalyst corrosion.

## Data Availability

The authors declare that materials and date are promptly available to readers without undue qualifications in material transfer agreements. All data generated in this study are included in this article.

## Abbreviations

BQ: *p*-benzoquinone
DRS: UV-vis diffuse reflectance spectroscopy
EDX: Energy dispersive X-ray spectrometer
HR-TEM: High-resolution transmission electron microscopy
IPA: Isopropanol
RhB: Rhodamine B
SEM: Scanning electron microscopy
TEM: Transmission electron microscopy
TEOA: Triethanolamine
XPS: X-ray photoelectron spectroscopy
XRD: X-ray diffraction
